# Mesothelial to mesenchyme transition as a major developmental and pathological player in trunk organs and their cavities

**DOI:** 10.1038/s42003-018-0180-x

**Published:** 2018-10-16

**Authors:** Tim Koopmans, Yuval Rinkevich

**Affiliations:** Comprehensive Pneumology Center, Institute of Lung Biology and Disease, Helmholtz Zentrum München, Munich, 81377 Germany

## Abstract

The internal organs embedded in the cavities are lined by an epithelial monolayer termed the mesothelium. The mesothelium is increasingly implicated in driving various internal organ pathologies, as many of the normal embryonic developmental pathways acting in mesothelial cells, such as those regulating epithelial-to-mesenchymal transition, also drive disease progression in adult life. Here, we summarize observations from different animal models and organ systems that collectively point toward a central role of epithelial-to-mesenchymal transition in driving tissue fibrosis, acute scarring, and cancer metastasis. Thus, drugs targeting pathways of mesothelium’s transition may have broad therapeutic benefits in patients suffering from these diseases.

## Abstract (web summary)

Tim Koopmans and Yuval Rinkevich review recent findings linking the mesothelium’s embryonic programs that drive epithelial-to-mesenchyme transition with adult pathologies, such as fibrosis, acute scarring, and cancer metastasis. They highlight new avenues for drug development that would target pathways of the mesothelium’s mesenchymal transition.

## Introduction

Our internal organs and cavities are lined by a single continuous layer of epithelial cells known as the mesothelium that is derived from the embryonic mesoderm. It is the largest epithelial organ in the adult mammalian body^1–3^. The mesothelium covers several body cavities and their internal organs, including (but not limited to) the pericardium, encasing the heart, the pleural cavity, encasing the lungs, and the mesentery and peritoneum, encasing the various abdominal organs. The mesothelium also surrounds the internal reproductive organs in both males and females. The mesothelial membranes consist of a parietal (lining the body wall) and visceral (lining the internal organs) layer. The space in-between is filled with fluid, which acts to accommodate organ movement and decrease friction. Other well-known functions include protection against bacterial infections and their disseminations within the cavities, and to provide direct passage between the cavities and the internal organs^4^ (Box 1).

All vertebrate animals have a coelomic cavity that separates the outer and inner components of the body. It is formed as a result of a binary division of the lateral plate mesoderm. Through this division the coelom is lined by two different, but continuous tissue components^5^. During organ development, a cell population within these tissues acquires epithelial features, baso-apical polarization, and a basal lamina. It is at this point that we refer to these cells as the coelomic epithelium, the embryonic precursor of adult mesothelium. The coelomic epithelium significantly contributes to organ development, in particular by undergoing epithelial-to-mesenchymal transition (EMT). Through EMT, the coelomic epithelium contributes various cell types into the developing and growing organs, including fibroblastic cells and smooth muscle cells^6^. We and others have identified some of its distinguishing markers^6^, and used it to lineage trace the mesothelium’s embryonic and adult precursors. A subset of these cells are the likely self-renewing stem and progenitors of the underlying organ fibroblasts and smooth muscle^6^. In adult life, the same pathways that are involved in EMT during organ development and growth reappear during injury and organ disease, such as infarctions, ischaemia, fibrosis (developing within organs), adhesions (developing in-between organs, tethering them to one another), and cancer. We propose here that trunk organ injury and disease can be best understood in light of re-emergences of these cellular and molecular EMT programmes specific to mesothelial development. To illustrate this point we describe four organ systems: the heart, liver, gonadal system, and the lungs. We review the current literature on mesothelial cell involvement in normal development and growth of these organ systems, where a remarkable genetic overlap exists between different coelomic epithelial tissues. We then summarize the literature on injury and disease of these organ systems in adult life and show that these diseases recapitulate the normal embryonic expression of coelomic epithelial genes. We conclude with further examples of involvement of these genes in pathology of the abdominal wall (peritoneum) and in mesothelium-related cancers. As the pathologies across these organ systems are essentially characteristic of uncontrolled EMT progression, we deliberate on the impetus to exploit EMT for therapeutic gain and pose some pending questions and challenges relevant for the field.

### Box 1 The different functions of mesothelium

The mesothelium provides a lubricating non-adhesive surface that constitutes a frictionless interface between mobile adjacent organs and/or their cavities^4^. This is achieved through the synthesis of various phospholipids, glycosaminoglycans, and proteoglycans, which endows the mesothelium with a protective glycocalyx and selective permeability properties^221,222^. Frictional injury is additionally prevented by microvilli on the external surface of mesothelial cells.

Microvilli also increase the absorption of solutes and trap water and serious exudates^4^, which are actively transported across the mesothelium to regulate volume and pressure of the cavities. The serosal fluid generated by the mesothelium acts as a niche and harbours various immune cells and metabolites that sustains immune responses^223–227^. Cells are constantly circulating across abdominal organs and towards lymphatics through peristalsis^228^, enabling them to respond rapidly to injury or infection^229,230^.

Immune responses are further facilitated by the mesothelium itself through their expression of multiple pattern recognition receptors. These recognize carbohydrates and lipopolysaccharides on the surface of microbial pathogens, and in response mesothelial cells secrete inflammatory cytokines and cell-adhesion molecules, promoting immune cell influx and microbial clearance^231^. On a tissue level, the mesothelium of the greater omentum promotes immunity to peritoneal antigens by encapsulating a highly vascularized parenchyma of fatty tissue and multiple immune cell aggregates called milky spots, which serve as unconventional, secondary, lymphoid organs^232^. Milky spots dynamically increase in number and size during chronic inflammation^233^, and provide a source of antibody production for adaptive immunity^232,234^.

## Mesothelial cell EMT involvement in healthy organ development and growth

### Heart development

**Fig. 1:**
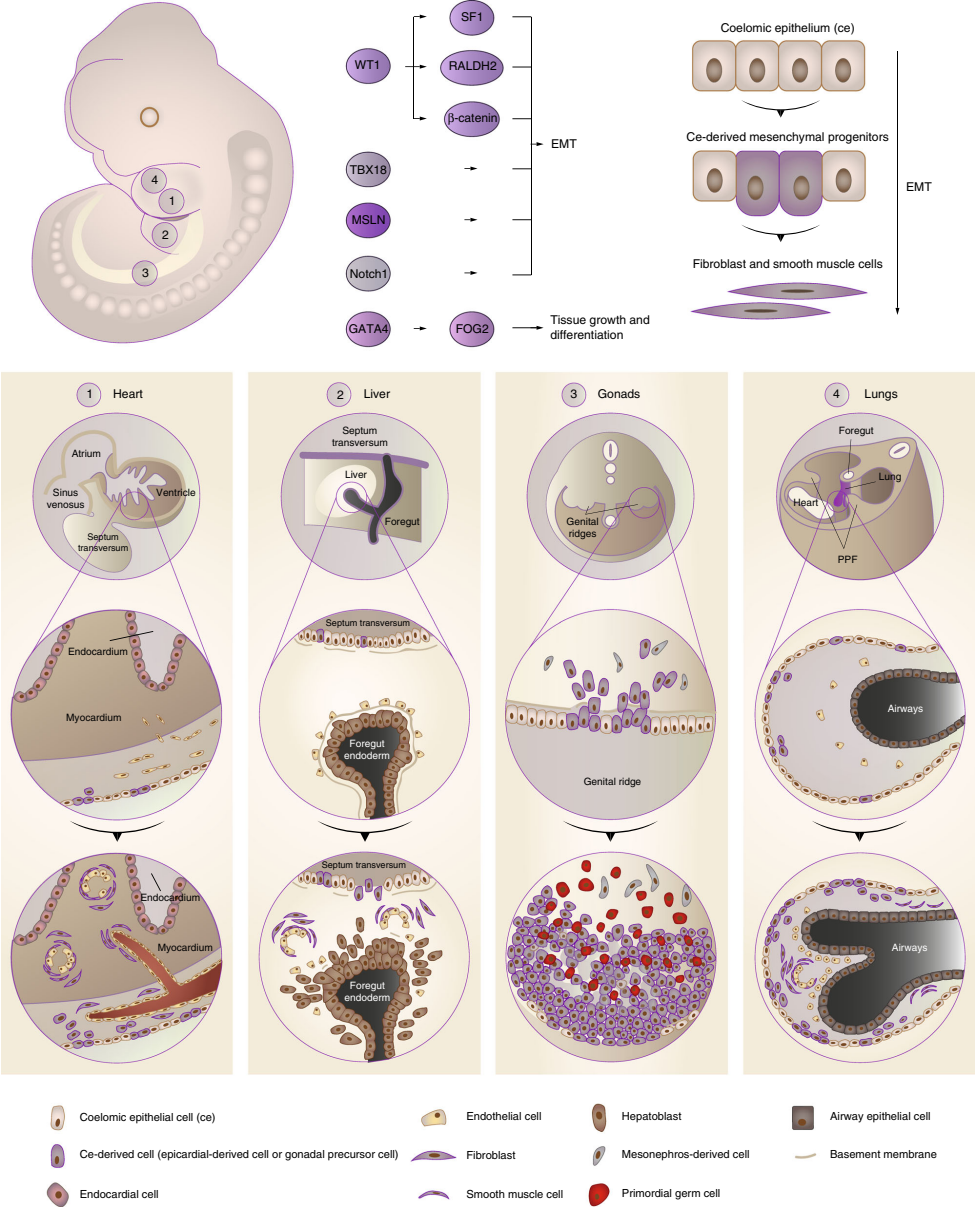
Mesothelial involvement in embryonic development. Coelomic epithelial cells provide the bulk of fibroblastic and smooth muscle lineages of the body and critically support EMT during embryonic development of different organs. A common set of genes, in particular WT1, TBX18, MSLN, Notch1, GATA4, and their downstream effectors underlie these EMT programmes to regulate mesenchymal differentiation. Four examples are given: (1) heart, (2) liver, (3) gonads, and (4) the lungs, showing how the coelomic epithelium expands and ingresses to form tissue-specific fibroblasts and smooth muscle lineages (displayed as purple) by virtue of EMT. For further details, see the main body of text

The coelomic epithelium that covers the developing heart is derived from a group of progenitor cells near the venous pole of the heart known as the proepicardial organ, which in turn comes from a thick mass of cranial mesenchyme called the septum transversum. The precursors of the coelomic epithelium migrate to form a layer of tissue between the pericardium and the heart, termed epicardium. A subpopulation of these cells proliferates and undergoes EMT that allows them to invade the developing heart and colonize its internal spaces as subendocardial fibroblasts, and by encircling the developing coronary blood vessels, where they develop into smooth muscle cells^7,8^ (Fig. 1). Indeed, several independent lineage-tracing experiments using epicardial-specific Cre-LoxP transgenes, e.g. mesothelin (*Msln*), T-box transcription factor 18 (*Tbx18*), and Wilms’ tumour 1 (*Wt1*), have demonstrated that epicardial cells can differentiate into coronary smooth muscle cells, and fibroblasts (including pericytes)^6,9–13^. The importance of the epicardium in heart development has been further demonstrated with epicardial knockout models. From these studies, a number of genes have been identified that highlight epicardial EMT as a crucial factor in heart morphogenesis. TBX18 over-expression promotes EMT in cultured epicardial cells by inducing the expression of Slug^14^. The role of the transcription factor WT1 in epicardial development has been described in more detail. WT1 binds directly to the Snail and E-cadherin promoter sites, thereby either promoting or repressing transcription respectively^12^. Mice with a *Wt1* deletion in the epicardium develop peripheral oedema, pericardial haemorrhage, and heart wall thinning^12,15^ resulting from impaired EMT^16^. In line with this, epicardial *Wt1* knockouts have reduced epicardial expression of the key EMT activators Snail and Slug, and show reduced expression of the mesenchymal marker Vimentin^12,16^. This is paralleled by increased expression of the epithelial markers E-cadherin and cytokeratin^17^. WT1 is an upstream regulator of the canonical WNT signalling pathway that leads to activation of β-catenin. Epicardial-specific β-catenin-null mice have disturbed epicardial EMT, blunted invasion of the heart muscle, failed expansion of the sub-epicardial space, and impaired differentiation of epicardial cells into coronary smooth muscle^16,18^. EMT also fails in cardiac fibroblasts (that remain in the epicardium) in the absence of the β-catenin-binding partner TCF21^19,20^. Another WNT protein, WNT5A has also been implicated as an epicardial factor that promotes compact heart muscle growth^21^. Both *Wnt5a* and *Wt1* mouse knockout hearts have thin compact heart muscle^16^. Another epicardial gene whose expression is dependent on WT1 and that is important for heart development is retinaldehyde dehydrogenase 2 (*Raldh2*), encoding the enzyme that controls retinoic acid availability. *Wt1* knockouts show reduced RALDH2 expression, and like *Wt1* knockout embryos, *Raldh2*^−/−^ embryos, treated with exogenous retinoic acid to bypass early embryonic lethality^22^, exhibit myocardial hypoplasia and coronary vessel abnormalities. Retinoic acid supplementation also works to restore embryonic EMT and viability of *Wt1* knockout hearts, suggesting a functional link between WT1 and RALDH2 (refs. ^16,23^). Like WT1, retinoic acid also regulates differentiation of epicardial cells^24^, thus the contribution of RALDH2 deficiency to the WT1 knockout phenotype is likely multifactorial. Differentiation of epicardial cells is also regulated by retinoid X receptor alpha (RXRα), as epicardial-selective knockouts have impaired EMT that disrupts coronary arteriogenesis and obviates myocardial compaction^25^. In this regard, other genes act in close cooperation with epicardial EMT. *Gata4* (refs. ^26,27^) and its co-factor Friend of GATA 2 (*Fog2*)^28,29^ deficiency results in embryonic death in mice due to vascular defects and myocardial thinning^26,30,31^. Finally, epicardial Notch signalling is also directly involved in heart development. Mouse mutants defective in the Notch effector RBPjκ show premature EMT in the form of ectopic patches of alpha-smooth-muscle-actin (α-SMA) at the venous pole of the heart, but show no alterations in expression of WT1, TBX18, GATA4, and RALDH2 (ref. ^32^). This may indicate an EMT programme independent from WT1. *Wt1*-specific Notch1 knockouts display severe defects due to defective EMT, leading to embryonic lethality in some cases. Like *Wt1* mutants, these mice develop a severe reduction and disorganization of the coronary vascular plexus, and dramatic reduction in the number of smooth muscle cells in the compact myocardium^32^. Thus, different Notch effectors may regulate different aspects of EMT, depending on the stage of development. Collectively, epicardial EMT is controlled by a plethora of factors, where WT1 appears to be the master regulator that sits atop of a hierarchy of gene loci required for EMT and proper heart formation.

### Liver development

In mice, the liver is initially formed as an out pocket of the foregut endoderm, but subsequently invades the surrounding septum transversum (which also contributes to heart development) and forms a hepatic cord to complete its development. Cells of the septum transversum proliferate actively to cover the hepatic lobes and migrate inward to generate mesenchymal lineages^33^. Lineage-tracing studies with *Msln*^CreERT2^ (ref. ^6^), *Wt1*^CreERT2^ (refs. ^34,35^) and *Gata4*^Cre^ (ref. ^36^), combined with reporter lines have shown that coelomic epithelium progenitors give rise to hepatic stellate cells, smooth muscle cells and fibroblasts that incorporate into the sinusoidal walls of the liver lobes (Fig. 1). The same molecular programmes that induce the epicardium to undergo EMT and to contribute cellular derivatives during heart development are observed in the developing liver. Consequently, many of the transgenic lines where EMT genes are knocked out show liver phenotypes in addition to heart phenotypes. For example, *Wt1* and *Rxra* knockout mice fail to properly generate mesenchymal cell lineages, resulting in liver defects through a reduction in liver size and lobe abnormalities^37^. Similarly, coelomic epithelium from the septum transversum requires *Wt1*-driven WNT/β-catenin^38^, and loss of β-catenin in the coelomic epithelium and stellate cells expressing TWIST2 causes abnormal activation of stellate cells in embryonic livers^39^. In *Gata4*-deficient mice, the liver bud is able to form a pseudo-stratified epithelial liver, but fails to expand^27,36,40^. Both *Gata4* (ref. ^36^) and *Wt1* (ref. ^37^) knockouts have an increased activation state of hepatic stellate cells, with abnormally high expression of α-SMA and deposition of ECM, reminiscent of myofibroblastic differentiation. This suggests that next to EMT, lineage-specific programmes may be regulated through GATA4 and WT1 (ref. ^41^).

### Development of the reproductive (gonadal) system

The adrenal cortex and gonads share a common developmental origin, the adrenogonadal primordium. In mice, the adrenogonadal primordium can be first detected as a thickening of the coelomic epithelium at approximately E9.5 (refs. ^42,43^). By E10.5, the adrenogonadal primordium splits into the adrenal and gonadal primordia that will continue to differentiate separately. The gonadal primordium, also known as the genital ridge, is initially bi-potential, and begins development through the increased proliferation of the coelomic epithelium (Fig. 1). This increased proliferation leads to the transformation of the monolayer epithelium into a dense and pseudo-stratified layer. Lineage-tracing studies using cell-permeable dyes^44^ or genetic Cre lines (*Wt1* (refs. ^45,46^), *Tbx18* (refs. ^47,48^), or steroidogenic factor 1 (*Sf1*)^49^) have shown that following these events, the basement membrane underneath the coelomic epithelium disintegrates, which allows the proliferating cells of the coelomic epithelium to undergo EMT, and to ingress and form a cluster of SF1^+^ gonadal somatic precursor cells^50,51^, which later give rise to the supporting Sertoli cells of the testis cords and seminiferous tubules^52,53^, or the follicular (or granulosa) cells of the ovarian follicles^54,55^. The supporting cells are not the only cells derived from the coelomic epithelium in the primitive gonads of mice. Cell-tracking experiments have shown that the SF1^+^ gonadal precursor cells generate the testicular peritubular myoid cells (PMCs, the smooth muscle cells surrounding the seminiferous tubules in the testis) through EMT and the steroidogenic Leydig cells and Theca cells in testis and ovaries, respectively^44,46–48,53,56–58^. The molecular mechanisms driving coelomic epithelium transformation into the genital ridge have not been laid out as comprehensively as for the embryonic heart or liver, but a similar gene signature in gonadal tissues can be observed around this time. Like the heart and liver, in the developing gonads *Wt1*, *Gata4*, *Tcf21* and *Tbx18* are one of the earliest activated genes, present throughout the whole urogenital ridge. Their expression is quickly followed by *Sf1*, which remains restricted to the genital ridge^59^. *Tbx18* null mice appear to have defective EMT in that they develop ectopic patches of fibroblasts along the developing gonads, and *Tcf21* knockouts lack a distinct mesenchymal compartment^60,61^. GATA4 is thought to be most important in the undifferentiated precursor population giving rise to the genital ridge. *Gata4* null mice do not develop a genital ridge at all, and the expression of SF1, but not WT1, is significantly decreased^59^. Similarly, *Fog2* mutants have abnormal gonads through impaired thickening of the genital ridge^62^. Mice lacking functional WT1 initiate gonad formation by thickening of the coelomic epithelium through active division, but the genital ridge quickly regresses before it is completely formed^15,45^. There is further evidence to suggest that WT1 regulates SF1 in the undifferentiated genital ridge^63^. Consequently, disruption of SF1 expression results in delayed and decreased EMT, disrupted cell ingression during the formation of the genital ridges, and consequently fewer SF1^+^ gonadal precursor cells and gonad size^64^. A microarray screen for WT1 wildtype and knockout E11.0 embryos highlighted *Muc16* as the top gene with regard to the coelomic epithelium, which was completely absent in knockout tissues^65^. Mucin 16 is highly expressed in mesothelial cancers, and has been shown to promote EMT, in part through interaction with MSLN^66,67^. Thus, similar to heart and liver development, WT1 appears to regulate EMT in the developing gonads, in part through SF1, while GATA4 closely cooperates alongside WT1 to drive initial formation of the genital ridge. In XY mice, knocking out *Wt1* after sex determination results in disruption of developing seminiferous tubules through the aberrant differentiation and development of Sertoli cells^68,69^. Sertoli-specific *Wt1* mutants also show abnormal PMC and Leydig development^70^. Interestingly, the precursors of Sertoli cells express only very low amounts of SF1, despite a high abundance of WT1, and it has been shown that after sex determination, SF1 is repressed by WT1 through direct binding to the promoter region^71^. In the absence of WT1, high SF1 expression leads undifferentiated somatic cells to develop into steroidogenic cells of the gonads (Leydig and Theca cells in the testis and ovaries, respectively)^69,71,72^, leading to structural impairments. These findings are consistent with the natural progression of SF1 expression throughout the developing gonads. In XY mice, SF1 expression starts to cease in the coelomic epithelium covering the genital ridge, but remains expressed in gonadal precursor-derivatives (pre-Sertoli cells and Leydig cells) around E12.5, and later persists only in Leydig cells^73^. Thus, similar to the Stellate cell of the liver, after sex determination WT1 acts to maintain lineage specification in the gonads. This may explain why WT1 expression is maintained in ovarian granulosa cells and testicular Sertoli cells of adult mice^15^; however, the origin of gonadal cell fates and the mechanisms of their differentiation still require further studies.

### Development of the lungs

In mouse embryos, the lung primordium emerges from the ventral foregut around E9.5, consisting of a simple tube of endodermal epithelium surrounded by splanchic mesodermal mesenchyme that is lined by a coelomic epithelium layer. This tube then branches to form the pulmonary sacs^74^. Anterior to the developing lungs are bilateral folds of tissue which extend from the body wall, known as the pleuropericardial membranes, which grow towards the midline, each encapsulating a common cardinal vein and phrenic nerve. They are further stretched when the initially straight embryonic heart tube is transformed into a helical loop, to finally fuse with the root of the lungs to close off the remaining communication between the pericardial and pleural spaces around E13.5 (ref. ^75^) (Fig. 1). Because of the contiguous nature of the heart and pleura, many gene pathways are shared between these organs. WT1, TBX18, RALDH2, β-catenin, Notch1, and GATA4, at some stage in lung development, are all selectively expressed in coelomic epithelium cells surrounding the lung buds or pleuropericardial folds^76–81^. Lineage-tracing studies have confirmed that by E11.5, WT1+^78,79,82–84^, MSLN+^6^ and Notch1+^79^ coelomic epithelium cells covering the lungs migrate inward to give rise to α-SMA+ fibroblast and smooth muscle cells that make up both arteries and veins in the vascular wall, or those positioned circumferentially around the airways. These results have been extended towards the postnatal lung using inducible Cre lines, indicating that the coelomic epithelium contributes to these lineages even after birth^82^. Equally, WT1+ descendants from the pleuropericardial folds generate the mesenchyme surrounding the cardinal veins adjacent to the growing lungs^85^. Knockout studies have corroborated these findings. Defects in *Gata4* or its co-factor *Fog2* cause lung lobulation abnormalities, which can be attributed to growth delay and abnormal branching morphogenesis^81,86^. Internally, *Gata4* mutants show dilated distal airways and patches of thickened mesenchyme, characterized as ectopic expression of α-SMA in the mesothelial and submesothelial layer^87^. Similar observations have been made in the pleura of mice harbouring a mutant of P300, another known co-factor for GATA4 (ref. ^88^). These results suggest defects in GATA4 expression perturb coelomic epithelium-driven EMT during lung morphogenesis, presumably through impaired signalling from the mesothelium to the underlying endoderm. Similarly, coelomic epithelium-selective (*Dermo1*+) β-catenin mutants show impaired lung growth as early as E12.5, primarily through reduced mesenchymal expansion^89,90^, and defective Notch signalling in *Dermo1*+ RBPjκ mutants results in impaired vascular, but not bronchial smooth muscle differentiation^79^. Systemic *Wt1* knockout embryos show severe growth defects in the lungs, displaying an irregular rounded-shape and abnormally fused lobes^15,78^. *Raldh2* mutant mice^91,92^ or lung explants treated with the pan-RXR inverse agonist BMS493 have perturbed lung bud formation^93^. *Wt1* and *Raldh2* mutants initially form a pleuropericardial fold, but do not fuse with the coelomic epithelial lining of the lung hilus^75,85^, and *Tbx18* knockout mice fail to develop the pleuropericardial membranes at all^75^. In this regard, drivers of EMT have been shown to be essential in the underlying pathology. *Wt1* knockouts maintain an intact basal lamina under the pulmonary coelomic epithelium, and show strongly reduced vimentin immunoreactive cells in the lung mesenchyme, indicative of impaired mesothelial EMT^78^. Several downstream targets have been shown elementary in this process. Embryonic lung explants exposed to CCSFE to label pleural coelomic epithelium show extensive EMT after 48 h, which can be further controlled through TGF-β or Notch signalling modulation^79^. Another putative regulator of mesothelial EMT is Enhancer of zeste homologue 2 (EZH2), a histone methyltransferase that in mesothelial cancers is known to repress EMT^94^. *Wt1*-dependent EZH2 knockouts display uncontrolled EMT in the coelomic epithelium, and develop multiple heart and lung abnormalities by generating ectopic patches of smooth muscle around E14.5 (ref. ^95^), originating at the proximal end of lung lobes, and then spreading out distally around E18.5. These patches co-express smooth muscle protein 22 (SM-22)α and α-SMA, indicative of a smooth muscle phenotype, and are closely associated with WT1+ cells at the periphery of the lung.

## Injury and disease in adult life: recapitulation of coelomic epithelial genes

### Pathology of the heart

The mesothelium covering the heart (epicardium) is directly involved in several types of heart diseases, an example being myocardial infarction. Induction of myocardial infarction in mice results in re-activation of the epicardium and the re-expression of several embryonic epicardial genes, such as *Raldh2*, *Wt1*, *Gata4*, and *Msln*^96–99^. The re-expression of embryonic genes is typically confined to the lesion site, where the epicardium progressively expands and undergoes EMT to generate cardiac fibroblasts within the vicinity of the infarct^96,100–102^. These areas are rich in expression of mesenchymal cell type markers, including fibroblast-specific protein 1, pro-collagen I, collagen III, fibronectin, α-SMA, SM-22α, and smooth muscle myosin heavy chain^96^, indicative of fibroblasts that are actively depositing extracellular matrix proteins. Epicardium-specific inhibition of these coelomic epithelial genes significantly diminishes cardiac fibrosis. For example, *Wt1*^Cre^;*Ctnnb1*^flox^ mice are null for β-catenin expression in the epicardium, and show minimal signs of epicardial expansion and EMT when subjected to ischaemia reperfusion injury^101^. In mammals, expression of a dominant-negative CCAAT/enhancer binding protein, which in essence is upstream of and controls the transcription of *Raldh2* and *Wt1* in adult mouse epicardium, can instil a cardioprotective effect and partially prevent myocardial fibrosis after ischaemia reperfusion^97^. In spite of the fibrotic potential of injured mesothelium of the heart, its presence is probably required for normal wound repair. In adult zebrafish, a species with robust regenerative potential after injury, genetic depletion of the epicardium after experimentally resecting parts of the heart ventricle inhibits cardiomyocyte proliferation and delays muscle regeneration^103,104^. Similarly, disruption of thymosin β4-mediated paracrine signalling towards the epicardium impairs smooth muscle cell development in mice, and its expression facilitates vascular repair following myocardial infarction^105,106^. It is worth mentioning that not every cardiac injury model shows evidence for epicardial EMT, for example, in the pressure overload model through constriction of the ascending aortus^107,108^. However, this model primarily results in perivascular fibrosis, with no signs of elevated WT1 or TBX18 expression in the epicardium^109^, and thus likely originates from a different, possibly endothelial source^107,108^.

### Pathology of the liver

The mesothelium covering the liver is comprehensively involved in liver pathologies. Different hepatic injury models that exhibit varying degrees of fibrosis show varied involvements of the hepatic surface mesothelium, or hepatic stellate cells (HSCs), which are derived from mesothelium. In fact, different injury models show diverged severities of fibrosis that stem from the level of activation of the mesothelium and HSCs. Lineage-tracing studies have confirmed an active role of the surface mesothelium as the cell-of-origin of HSCs and myofibroblasts in response to carbon tetrachloride, which can be inhibited through antagonism of TGF-β signalling^110^. Similarly, activated portal fibroblast (the predominant source in cholestatic liver injury induced by bile duct ligation) exhibit a high expression of MSLN upon bile duct injury, further suggesting that these cells originate from a mesothelial source^34,111^. Consequently, MSLN-deficient mice are less susceptible to liver fibrosis compared to wild-type mice^112^, and conditional ablation of mesothelial-derived MSLN^+^ portal fibroblasts in bile duct-injured mice show significantly reduced liver fibrosis^113^. The mechanism by which MSLN promotes fibrosis presumably acts through its antagonistic actions on a TGFβR1 inhibitory complex called thymocyte antigen 1 (ref. ^113^). Like the heart, the mesothelium also supports liver regeneration upon injury. The mesothelium-derived paracrine factors MDK and PTN facilitate hepatocyte growth following hepatectomy^114,115^, analogous to the mesothelium’s stimulatory and regenerative role during intestinal crypt^116^ and embryonic heart regeneration^117^. Conditional *Gata4* knockout mice have a high degree of liver fibrosis when treated with subcutaneous injections of carbon tetrachloride, inducing hepatoxic liver injury^36^. Chronic exposure in GATA4-deficient mice results in increased liver hydroxyproline content, infiltrating CD45^+^ cells, and serum alanine aminotransferase and aspartate aminotransferase levels compared to control livers^36^, all indicative of fibrosis associated with progressive liver failure. These findings likely indicate that mesothelial expression of GATA4 inhibits liver fibrosis through its effects on lineage maintenance in Stellate cells.

### Repair of the (female) reproductive system

The ovaries regenerate their surface mesothelium (also called ovarian surface epithelium, OSE) throughout postnatal life. Rupturing of the follicles that follows each ovulatory cycle is followed by complete regeneration of the mesothelium without fibrosis. In spite of this, ovulatory wound repair is nonetheless associated with mesothelial EMT that temporarily permits displaced OSE to assume a mesenchymal phenotype within the ovarian cortex and migrate actively towards the ovulatory wound^118–121^. Additionally, the EMT regulator TGF-β1 is highly expressed in the serosal fluid around the ovaries in response to ovulation, and significantly increases the sphere-forming efficiency of OSE progenitor cells, further suggesting EMT is a requisite for the maintenance of OSE integrity^122^. It has been proposed that discrete regions within the OSE may serve as cell sources that replenish or close the ovulatory wounds^123^, and several markers have been identified^124,125^, including lymphocyte antigen 6 complex, locus A (LY6A), leucine-rich repeat-containing G-protein coupled receptor 5 (LGR5), and RALDH (WNT target genes). For example, a subset of the OSE expresses LGR5 that serves to maintain OSE homoeostasis and act as a cell-of-origin for new OSE during the ovulatory cycle^121,126^. The exact location of LGR5^+^ cells is still under debate, as to whether they are restricted to the ovary hilum^121^, or are more widespread, and respond locally to rupturing follicles by actively proliferating^126^. Nonetheless, these findings suggest that the activity of LGR5+ mesothelial cells are most likely stem cells that are tailored to meet the specific growth requirement of the local OSE. The integrity of LGR5+ cells is likely maintained by active WNT signalling, as these cells secrete WNT4 and express several WNT target genes such as TROY and inhibitor of DNA binding 2 (ref. ^126^). High RALDH expression has also been identified in the OSE transitional zone, which constitutes approximately 5% of the total OSE population. In vitro studies have shown this cell subset has higher capacities to generate cell clones as compared to RALDH- cells, and similar to LGR5+ cells, these cells exhibit a greater degree of proliferation after ovulation^121^. It is unclear at this point whether these cells represent the same cell population, and importantly, whether these genes are involved in regulating mesothelial EMT to drive ovulatory wound repair.

### Pathology of the lungs

**Fig. 2:**
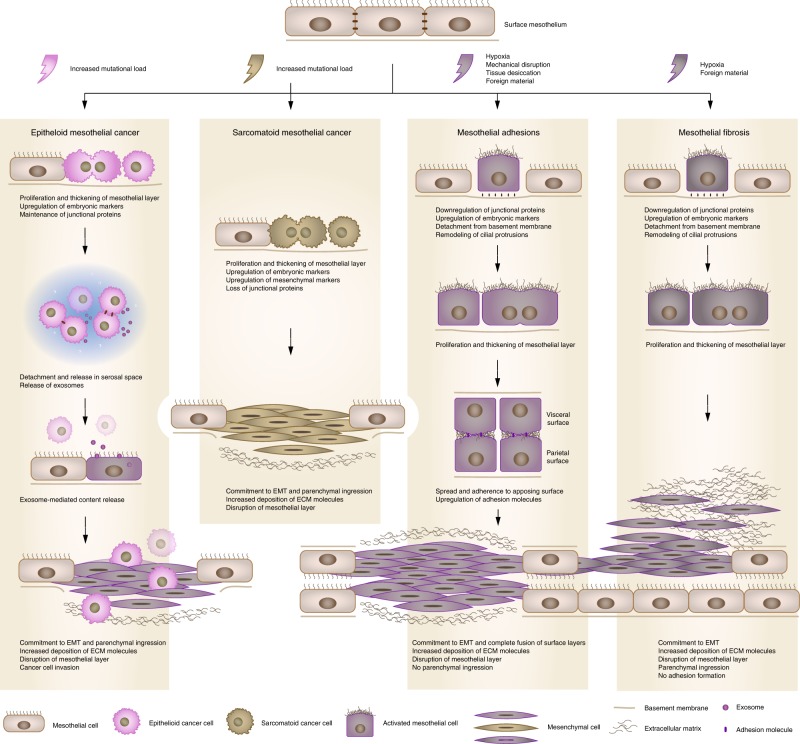
Fibroblastic lineage commitment is a common mechanism driving mesothelial pathologies. Many, if not all mesothelial pathologies ultimately converge on tissue fibrosis through the mesothelium’s ability to undergo EMT and generate fibroblastic lineages: (1) Epithelioid mesothelial cancers invade through their ability to influence resident mesothelium, in part by secreting exosomes, which then undergoes EMT and forms a cancer niche. (2) Sarcomatoid mesothelial cancers undergo EMT themselves to ingress in the parenchyma. (3) Mesothelial adhesions are similarly derived from EMT through various insults, which generates surface scarring that can spread to apposing organs and glues them together. (4) Damage to the surface mesothelium does not always result in adhesions, primarily when fibrosis progresses into the interiors of the (in most cases visceral) organs

There are two scarring pathologies of the lungs in which pleural mesothelium is known to be involved: pleural fibrosis (PF), and interstitial lung disease. PF manifests either as discrete localized fibrotic lesions, or more diffusely organized pleural fibrotic patches, which in some cases result in fusion of the pleural membranes (Fig. 2)^127^. Both parietal and visceral layers can be affected, but is generally only clinically significant when it involves the viscera^128^. Various etiologies can account for PF, from pleural effusions (e.g. rheumatoid, tuberculous, or uraemic pleurisy, bacterial empyema, retained hemothorax), to particle or compound exposures (e.g. asbestos, carbon nanoparticles, medications) or secondary malignancies^128^. Experimental models to study PF have relied mostly on variations of the bleomycin mouse model. In mice receiving intraperitoneal or intratracheal bleomycin, increased expression of the mesothelial markers calretinin and WT1 combined with TGF-β1-Smad2/3 is observed in the pleura and underlying parenchyma of mice^129,130^. Consequently, mice treated with the TGF-βRII targeting miRNA miR-18a-5p have reduced (sub-)PF after bleomycin administration^131^. Strikingly, intratracheal bleomycin combined with carbon nanoparticles result in a more persistent and severe pulmonary response, including adhesions and severe and progressive (sub-)PF, preceded by pleural thickening and inflammation^132^. The engulfment of carbon nanoparticles by the mesothelium contributes to the severity of this model, driving rapid upregulation of α-SMA and release of MMP-9 and TGF-β1 in the pleural fluids^132,133^. A role for β-catenin signalling has been described in a model for pleural effusion, a known risk factor for PF. Mice subjected to *Streptococcus pneumoniae* develop empyema accompanied by α-SMA+ expression in the pleura, pleural thickening and reduced lung function, which can inhibited by GSK-3β antagonist 9ING41 (ref. ^134^).

Interstitial lung diseases represent another collection of heterogeneous fibrotic parenchymal disorders, of which idiopathic pulmonary fibrosis (IPF) is a more clearly defined subset. In line with the mesothelium as the site of origin, clonal analyses in IPF lungs have revealed the presence of a polyclonal, rather than a monoclonal population of fibroblasts. This suggests fibrotic foci are due to a reactive process responsive to local environmental stimuli, rather than a single malignant neoplasm growing through the lung. These foci are part of a continuous fibrotic reticulum that extends from the mesothelial surface into the lung parenchyma, identifying the mesothelium as the most likely source of origin (Fig. 2)^135^. Further evidence has been derived from mathematical models, in which increased stiffness of the visceral pleura, e.g., following EMT and matrix synthesis, is predicted to be key in driving mechanotransduction pathways to propel a fibroblast activation chain towards the parenchyma^136^. In support of this, the pleura and underlying parenchyma of human IPF patients show strong immuno-labelling for MSLN, WT1 or calretinin^130,137,138^, and when isolated and cultured display increased contractility and movement compared to healthy mesothelium^130^. The extend of labelling correlates positively with the degree of parenchymal fibrosis in patients^137^. Importantly, these markers are absent in other fibrotic diseases of the lung, such as chronic obstructive pulmonary disease or cystic fibrosis, in which the mesothelium plays no significant role. In vitro, pleural mesothelial EMT is controlled by a WT1–TGF-β1–Smad-2 axis^138^, which is further supported by mouse models. Adenoviral transmission of intrapleural TGF-β1 induces progressive PF of the viscera and underlying parenchyma, with strong cytokeratin and α-SMA in both tissue compartments^139^. Lineage tracing of these cells with WT1^GFP-Cre^ mice or GFP-labelled pleural mesothelium injected in the pleural space, combined with intratracheal recombinant TGF-β1 show strong GFP and α-SMA co-labelling in the lung parenchyma underneath the pleura^130,137^, indicating active migration of the mesothelium.

### Pathology of the abdominal wall (peritoneum)

Damage to mesothelium covering the three cavity walls or the internal organs induces the development of fibrosis on their surfaces that can attach to neighbouring healthy tissues, termed adhesions^140^ (Fig. 2). Adhesions are the most common side-effect of abdominal surgery that stems from mechanical trauma due to surgical tissue handling, ischaemia at incision sites, foreign bodies or tissue desiccation^141,142^. As well as after surgery, adhesions can result from inflammatory processes, from infections, or as an adverse response to dialysis^143^. Kidney dialysis takes advantage of the peritoneal surface mesothelium as a means of extravasation of plasma fluids from the blood. The dialysate solution, which contains solvents that are hyperosmotic, hyperglycaemic, and of low pH, gradually damages the exposed surface mesothelium and leads to its activation^144,145^. Exposure of the mesothelium to dialysis solution increases mesothelial cell death^146^, but also eventually leads to thickening of the mesothelium and generation of myofibroblasts and fibrous collagen bands at the injured surface^144,147^. Once fibrous bands initiate at the surface, the mesothelium never reaches complete regeneration, even months after cessation of dialysis^146^. More severe cases involve the development of encapsulating peritoneal sclerosis on the surface mesothelium that follows prolonged exposure to dialysis, chronic inflammation, or several other secondary acute intra-abdominal events. Similar to other mesothelial pathologies, the pathomechanism that leads to encapsulation is poorly understood. Organ encapsulation stems from thickening and fibrosis of the surface mesothelium and leads to a range of ailments, including obstruction and shortening of the bowels, abdominal pain, weight loss, malnutrition, sepsis, or death^148^. The level of encapsulation and thickening directly correlates with the level of mesothelial activation^149^, suggestive of a pathomechanism wherein mesothelial cells undergo varied extents of EMT in response to durations of dialysis^150–152^. Human peritoneal–intestinal adhesion tissues resulting from abdominal surgery show strong immuno-labelling for cytokeratin, calretinin, WT1, and α-SMA in the mesothelium and surrounding fibroblasts, suggesting these fibroblasts derive from mesothelial cells that have undergone EMT^153^. The advent of lineage tracing has confirmed these observations. Although WT1^+^ mesothelial cells have been reported to proliferate and actively repair the peritoneal surface upon injury^154^, various models of peritoneal fibrosis (i.e. hypochlorite, daily intraperitoneal injection of dialysate or mouse TGF-β adenoviral administration) have reported a mesothelial and submesothelial origin for myofibroblasts in peritoneal fibrosis^154,155^. Mechanistic studies have suggested TGF-β to be an active player in peritoneal EMT^156–158^. Conditional deletion of the *Tgfbr2* gene in WT1+ mesothelial cells, treatment with the TGF-β1-blocking peptide p144 or p17, or use of mutant mice deficient in Smad3 all significantly attenuate the fibrotic response in (both) visceral and parietal mesothelium in several peritoneal fibrosis models^153,155,159,160^. Accordingly, these events are parralelled by downregulation of EMT markers, such as Snail^153^. We have recently established a mouse model of abdominal adhesions, wherein ligating small areas within the peritoneum, combined with gentle localized abrasion generates adhesions between the body wall and intestine^161^. In this model, MSLN and WT1 are rapidly upregulated in the mesothelial layer, followed by active proliferation. Within 1 h mesothelial cells show early morphologic signs of EMT, extending cilial protrusions into the peritoneal space and subsequently detaching from the basement membrane and neighbouring cells (Fig. 2). Cell tracking studies confirmed a mesothelial origin for peritoneal adhesions. Remarkably, these changes are associated with a massive genome-wide transcriptional rearrangement (more than 8000 genes) within 24 h after injury. Early gene programmes involved upregulation of angiogenesis and hypoxia, followed by inflammatory responses, as well as cell cycle markers and the downregulation of adhesion molecules. Many markers that are highly expressed by the peritoneal mesothelium during embryonic development and with very low expression during normal homoeostasis of adult mice, e.g. MSLN, Uroplakin-1B, and WT1, all peaked at 24 h after injury. Genes associated with fibroblasts (fibroblast-specific protein 1 and α-SMA) were also highly upregulated, paralleled by spindle-shaped mesothelial cells, further suggesting active EMT.

### Pathology of mesothelium-derived cancers and cancer-associated fibroblasts

Mesothelial EMT is also linked with the progression of various tumours. The most well-known are malignant mesothelioma, a rare kind of cancer that originates from the visceral or parietal mesothelium of the thoracic, pericardial, or trunk cavities^162,163^, and ovarian cancer, particularly type II high-grade serous carcinoma. Mesothelioma most often originates in the pleura^164^, and is usually triggered by asbestos exposure^165^, or inherent germline mutations^166^, which can act synergistically^167,168^. It is broadly divided into three histological subtypes: epithelioid, biphasic (or mixed) and sarcomatoid, the latter occurring in approximately 10% of patients^169^. The prognosis for patients with mesothelioma is dismal, and patients with sarcomatoid mesothelioma have particularly poor outcomes compared to patients with epithelioid histology^169^. Ovarian cancer was initially thought to originate exclusively in the ovaries^170^, but the discovery of a possible precursor lesion in the fallopian tube fimbria provided evidence for possible multiple sites of origin^171–173^. Ovarian cancer, but also mesothelioma, spreads by disseminating from the primary site^174,175^, where cells enter the serosal fluid to be carried to secondary sites^176^. Both mesothelioma and ovarian cancer are highly aggressive, evolve rapidly, and are usually diagnosed in an advanced stage where curative surgery is no longer an option. Both OSE and fallopian tube fimbria-derived human tumours, as well as mesotheliomas, are characterized by high expression of Notch1 and MSLN or its derivative, serum soluble mesothelin-related peptide^175,177,178^, and cell culture studies have indicated MSLN is involved in driving cancer EMT^179^. Mesothelial-derived cancers are remarkably heterogeneous. Most metastasizing solid tumours must first invade the tumour stroma and access the vasculature. Therefore, an early EMT is necessary in order to adopt a motile, invasive phenotype. However, the unique trans-coelomic route of mesothelial-derived cancer cells creates an exceptional microenvironment. In contrast to most solid tumours, mesothelium-derived cancer cells exfoliate directly into the peritoneal cavity. Thus, early events in metastatic dissemination do not necessarily require a mesenchymal phenotype. As a result, both pleural and ovarian mesothelial cancer cells can display either epithelial or mesenchymal morphologies, or exist as an intermediate state. For example, sarcomatoid mesotheliomas resemble spindle-shaped cells that mimics the morphology of mesenchymal tumours, whereas epithelioid mesotheliomas represent a more epithelial-like morphology^169^. In sarcomatoid histology, expression of MSLN or WT1 is generally absent, concomitant with high activation of mesenchymal genes, such as *Loxl2* or *Vim*^180^. Conversely, epithelioid mesotheliomas retain epithelial features, such as expression of the junctional protein E-cadherin^181,182^, and express high levels of mesothelial markers, among which UPK3B, R-spondin 1, WT1, and MSLN. Genes necessary for EMT are lowly transcribed, such as *Cldn15* (refs. ^180,181^). Importantly, different EMT states can predict survival outcome in ovarian cancer patients, where highly mesenchymal cancers correlate with poorer disease-free survival, and epithelial cancers show better overall survival^183^. The ability of epithelioid mesothelioma to metastasize, presumably without requiring EMT, may represent a common behaviour of epithelial cancers^184,185^. It has been proposed that epithelial cancer cells can exploit non-malignant stromal cell types to develop cooperative invasion strategies. Cancer-associated fibroblasts are ideal stromal partners to enable collective cancer cell invasion^186–188^. A body of literature has suggested cancer-associated fibroblasts to originate from a local mesothelial source^189–192^, which has been confirmed with more recent lineage tracing studies. In a model where gastric cancer cells were implanted on the stomach surface, both resident mesothelium and implanted cancer cells mutually migrate towards each other after a rapid proliferative response by the mesothelial layer^193^. It was shown that the release of CD63+ exosomes by cancer cells largely dictated this response, where exosomes can always be found ahead of invading cancer cells and are actively incorporated into the infiltrated mesothelium (Fig. 2). Exosomes may contain growth factors used to directly modify the mesothelium, or matrixmetalloproteases (MMPs) to disrupt the basement membrane, and is not restricted to gastric cancer: CD63+_,_ CD44+^194^, MSLN+^195^, or MMP1+^196^ exosomes have been found in ovarian cancer as well. Isolated exosomes derived from cancer cells can induce proliferation^197^ and subsequent EMT^198^ in the peritoneum of mice when injected, promoting tumour survival. WNT/β-catenin and RALDH1 signalling appear elementary in these events, as they are highly upregulated in both gastric cancer and infiltrated mesothelium. Consequently, knockdown of these pathways effectively inhibits cancer invasion. A similar role has been shown for the peritoneum of mice. Gastric cancer cells injected into the peritoneal space quickly home to the peritoneum and start actively proliferating. In this study, like the stomach, invasion of cancer cells into the underlying stroma was always preceded by the rapid invasion of infiltrating mesothelial cells into the muscle layer. Infiltrated mesothelial cells covered a large area of the muscle layer, and were found abundantly within tumour nodules^192^. Lineage tracing confirmed that more than 90% of α-SMA expressing fibroblasts derived from mesothelium, indicating the occurrence of active EMT and a mesothelial origin of cancer-associated fibroblasts. These observations suggest that the mesothelium promotes cancer development by creating a niche that favours cancer cell invasion. Elevated expression of *Tks5* (a gene involved in the formation of actin-rich protrusions called invadopodia) was detected in mesothelial cells located on the invading edge of gastric cancer cells, which was abolished by neutralizing peptides^192^.

## Concluding remarks

Here we have presented evidence from various organ systems indicating that some of the most common trunk organ pathologies, such as infarctions, ischaemia, fibrosis, adhesions, and cancer, involve re-activation of genetic programmes that are normally expressed during the mesothelium’s embryonic EMT. These programmes initiate and regulate organ disease, rather than being a consequence of it. Moreover, the similarities in gene expression programmes across diverse organ systems and disease states suggest common roles of the mesothelium in driving organ pathophysiology. A deeper understanding of trunk organ diseases could thus emerge from studying the mechanisms underlying embryonic mesothelial EMT. Such a developmental focus might also identify much-needed new therapeutic avenues for trunk organ disease. For further research, three main aspects in mesothelium’s biology are particularly relevant: (1) intermediate stages of EMT, (2) common and organ-unique mechanisms of EMT, and (3) mesothelium’s cell type heterogeneity and their relation with EMT.

### Intermediate stages of EMT

Coelomic epithelium cells of the proepicardial organ need to go through a series of highly orchestrated steps to populate the embryonic heart primordium: (1) directed migration towards the heart surface, (2) controlled proliferation to increase cell numbers, (3) migration towards the inner compartments of the heart, and (4) gain of mesenchymal traits to adopt fibroblastic and smooth muscle characteristics. Not only do these stepwise events require alternating commitments to EMT and MET to sustain epicardial identity while also providing the mesenchymal lineages of the heart, they also require intermediate stages within the EMT spectrum. The plethora of genes described here that regulates these events, and the disparity between knockout lines with regard to their effects on EMT, highlights the complexity of (different stages of) EMT and the gene regulatory networks that are at play. Hence, it is important to consider that during scarring and wound healing in adult life, the same extent of heterogeneity may be expected, where cells may linger in intermediary stages between a full epithelial and mesenchymal state to promote wound healing or scarring. For example, studies on mesothelial ovarian cancers revealed they could be grouped in different compositions along the EMT spectrum^178,199,200^, which may reflect differently on their ability to migrate, adhere, invade or survive^201^. This stresses the importance to focus on the entire range of EMT to allow for a more dynamic interpretation of the fluidity and plasticity of this cell state. It may also provide more insight into the different scarring signatures recognized among mesothelial pathologies, e.g. why is the viscera sometimes more affected than the parietal surface, and why do some pathologies involve fibrous scars to exclusively develop on organ surfaces, whereas others extend into the parenchyma?

### Mechanisms of known EMT drivers

At present there is an underestimation of the complexity of WT1 signal regulation, and the networks in which it functions. This complexity includes alternative start codons, splice sites, and RNA editing that can theoretically give rise to 36 different proteins and many more potential dimers^202^. The creation of mice ablated for specific WT1 isoforms has provided direct evidence that *Wt1* splicing variants perform distinct functions in embryonic development^45,203^. Moreover, there may be gene elements that can influence the effects of WT1 isoforms. For example, WT1 drives EMT in the coelomic epithelium covering the heart, liver, and lung primordium through close cooperation with GATA4 under control of the G2 enhancer domain^204^. Contrary, in kidney development WT1 maintains epithelial integrity, where the *Gata4* gene is not driven by the G2 enhancer^205^. Thus, much work remains on the exact mechanisms that control mesothelial EMT, which likely requires a more systems-based approach. Establishing such gene regulatory networks and feedback loops will further our chance of developing successful therapies to reduce scarring and prevent fibrosis.

### Cell heterogeneity within the mesothelium

Single-cell transcriptomic studies have identified at least three epicardial cell subsets in the healthy adult heart of zebrafish^104^, and a sub-population of c-kit+ epicardials cells exists in the adult mouse epicardium^98,206^. However, although cultured epicardial cells from human and mouse adults are able to recapitulate at least part of the differentiation potential of their embryonic counterparts^117,207^, isolated adult epicardial cells of mice are not able to generate colony-forming units ex vivo^208^. As such, if the adult mesothelium of mice and humans contains a sub-population of cells with stem-like properties, they may exist in a dormant state^209^. A study in mice has recently revealed the presence of epicardial cell heterogeneity after subjecting hearts to models of myocardial infarction^210^. These cells expressed the progenitor markers CD90+, CD44+, CD105+, c-kit+, but were distinct from their embryonic counterparts^99,210^. Rather than an existing dormant progenitor state, stemness may be conferred to differentiated cells by changes in the local microenvironment in response to tissue injury. The idea that cell reprogramming can generate facultative stem cells emerged independently from several experimental models. For example, pancreatic ductal ligation causes broad degeneration of acinar cells^211^. However, the resulting survivor population is reprogrammed and exhibits embryonic multi-potency factors capable of partial tissue regeneration^212^. Whether this applies to adult mesothelial tissues remains to be explored. Further insight into the heterogeneity, the factors necessary to activate dormant or reprogrammable progenitor states, and how each of these populations contribute to EMT, scarring, and wound healing, will be future areas of research.

In recent years there has been a proliferation of studies on drugs targeting WT1 and MSLN, many of which are in advanced stages of clinical testing. Drugs include recombinant immunotoxin proteins, vaccines, monoclonal antibodies, and antibody-drug conjugates^213–215^. One example is Amatuximab, a chimeric high-affinity monoclonal IgG1/k antibody targeting MSLN, which elicits cellular toxicity against MSLN-expressing tumour cells, and reduces invasive capacity and tumour sphere formation^216^. Amatuximab improved median overall survival and was well tolerated in phase II clinical trials of patients with advanced pleural mesothelioma^217^. It would be important to determine whether patients receiving therapies targeting MSLN and WT1 have equally reduced risks of developing additional diseases such as ischaemia, chronic fibrosis, and adhesions. In this regard, other embryonic markers, such as WNT effectors, β-catenin, Notch1, and RALDH2, some of which are highly expressed in mesothelial cancers similarly warrant further investigation^218–220^. Clinical studies that target mesothelial cancers may help clarify the extent of co-morbidity with other mesothelial pathologies where EMT plays an important role, opening new clinical avenues through which our current understanding of the EMT programme and transitional events can be exploited and organ disease can be curtailed.
